# Reducing the Fatality Rate of COVID-19 by Applying Clinical Insights From Immuno-Oncology and Lung Transplantation

**DOI:** 10.3389/fphar.2020.00796

**Published:** 2020-05-26

**Authors:** Fatih M. Uckun

**Affiliations:** Department of Scientific Solutions and COVID-19 Task Force, Worldwide Clinical Trials, Wayne, PA, United States

**Keywords:** 2019-nCoV, acute respiratory distress syndrome, cytokine release syndrome, COVID-19, systemic capillary leak, acute lung injury

## Abstract

There is an urgent need to identify effective strategies that can stop or reverse the inflammatory process that causes acute lung injury, ARDS, and multi-organ failure in COVID-19. Adaptive clinical trials with parallel enrollment to different arms each evaluating a rationally designed combination modality could provide the foundation for the accelerated identification of effective and safe multi-modality treatment algorithms for COVID-19 pneumonia. This article summarizes the insights and lessons learned from clinical immune-oncology trials as well as lung transplantation that are informing the clinical development of promising new strategies aimed at reducing the fatality rate in COVID-19.

Fifteen to 30% of patients infected with SARS-CoV-2 (2019-nCoV), the causative agent of COVID-19, develop a severe form of pulmonary inflammation that results in acute lung injury and rapidly progresses to acute respiratory distress syndrome (ARDS) within 2 weeks, reminiscent of the ARDS caused by the pathogenic hCoVs SARS-CoV and MERS-CoV (Huang et al., 2020; Young et al., 2020). The observed high fatality rate of the acute lung injury caused by the new coronavirus (2019-nCoV) in high risk patient populations, such as elderly and patients with multiple co-morbidities, has prompted an intense search for treatments that can prevent a fatal outcome (Zumla et al., 2020). The documented systemic capillary leak and cytokine storm [also known as cytokine release syndrome (CRS)] in patients with 2019-nCoV–induced acute lung injury have been implicated in the immuno-pathology of ARDS and multi-organ failure associated with the severe forms of COVID-19 (Channappanavar and Perlman, 2017). Systemic capillary leak leads to intravascular fluid depletion with renal dysfunction, pulmonary edema, edema of interventricular septum, and myocardial dysfunction as well as viscous pericardial effusion further contributing to a decline of cardiac function (The National Heart, Lung, and Blood Institute Acute Respiratory Distress Syndrome (ARDS), 2006; Teachey et al., 2013; Garcia Borrega et al., 2019; Khadka et al., 2019). The standard supportive care for ARDS patients with systemic capillary leak or CRS is highly variable based on institutional preferences and includes combinations of supplemental oxygenation with progression to mechanical ventilation with low tidal volumes, fluid restriction, maintaining a high colloid osmotic pressure with blood products combined with diuretics, red blood cell transfusions to keep hemoglobin levels above 11 g/dl to improve the oxygen carrying capacity of the blood, use of low dose dopamine to improve renal perfusion, and sometimes the use of steroids. Unfortunately, fatality rate remains high with contemporary supportive care alone. An ongoing adaptive, randomized, double-blind, and placebo-controlled multi-center trial (ClinicalTrials.gov Identifier: NCT04280705) is designed to evaluate the safety and efficacy of novel antiviral agents in hospitalized adults diagnosed with COVID-19 as they become available. Preliminary results indicate that patients who received Remdesivir had a 31% faster time to recovery than those who received placebo (11 days vs. 15 days, p < 0.001), which prompted FDA to issue an emergency use authorization for potential COVID-19 treatment on May 1. Results also suggested a survival benefit, with a mortality rate of 8.0% for the group receiving Remdesivir versus 11.6% for the placebo group (p = 0.059). That being said, given the fulminant nature of this inflammatory process, it would seem highly unlikely that initiation of a specific antiviral therapy with Remdesivir (ClinicalTrials.gov Identifier: NCT04280705), hydroxychloroquine (Plaquenil) (ClinicalTrials.gov Identifier: NCT04318444), Favipiravir (ClinicalTrials.gov Identifier: NCT04310228), or other potential drugs under consideration for post-exposure prophylaxis after the onset of the pulmonary inflammation could significantly reduce the risk of ARDS or its mortality rate in symptomatic patients.

The use of convalescent plasma containing virus-specific antibodies has been shown to be highly effective in patients infected with SARS-CoV (Chen et al., 2020). A meta-analysis from 32 studies of SARS coronavirus infection and severe influenza showed a statistically significant reduction in mortality following CP therapy (Mair-Jenkins et al., 2015). Another investigational treatment being explored for COVID-19 involves the use of convalescent plasma containing antibodies to SARS-CoV-2 collected from recovered COVID-19 patients under an emergency IND according to expanded access provisions. The preliminary clinical proof of concept was provided by promising results in 5 COVID-19 patients with ARDS (Shen et al., 2020). Notably, their viral load declined within days of treatment and the clinical picture showed a substantial improvement with four patients who had been receiving mechanical ventilation and extracorporeal membrane oxygenation (ECMO) no longer needing respiratory support by 9 days after plasma transfusion (Shen et al., 2020). Investigators from over 20 institutions have formed a group, the COVID-1 Convalescent Plasma Project (CCPP19) to make the convalescent plasma therapy available to COVID-19 patients in critical condition. It remains to be seen if this empirical therapy could be made available to large numbers of patients and how effective it will be in patients with acute lung injury. Infection of receptor-bearing cells by pathogenic human coronaviruses is mediated by their spike (S) proteins. SARS-CoV infects cells expressing the receptor angiotensin-converting enzyme 2 (ACE2) (Shen et al., 2020; Tian et al., 2020). Notably, a soluble and catalytically inactive form of ACE2 potently blocked infection by SARS-CoV. Likewise, monoclonal antibodies to S-proteins have therapeutic potential as SARS-CoV entry inhibitors. Recently, a SARS-CoV–specific human monoclonal antibody was reported to effectively bind with nanomolar affinity the receptor binding domain (RBD) of the 2019-nCoV trimeric spike glycoprotein (Tian et al., 2020). Following the structural resolution of the 2019-nCoV RBD by cryo-electron microscopy, efforts are now underway to identify high affinity antibodies that recognize and block the ACE2 binding site of the 2019-nCoV RBD (Wrapp et al., 2020). However, these preclinical-stage antibodies will not be available for wide-spread use in COVID-19 patients in the immediate future and it is not clear if they could be beneficial beyond post-exposure prophylaxis. Therefore, there is an urgent need to identify effective strategies that can stop or reverse the inflammatory process that causes acute lung injury, ARDS, and multi-organ failure in COVID-19.

Interleukin-17 (IL-17) is involved in the pathophysiology of pulmonary diseases that are associated with neutrophilic inflammation (Ryzhakov et al., 2011). As a key contributor of the severe immunopathology of virus-induced potentially fatal pulmonary inflammation and ARDS associated with SARS coronavirus (SARS-CoV), MERS coronavirus (MERS-CoV), avian influenza virus, and respiratory syncytial virus (RSV), IL-17 has been shown to amplify proinflammatory host immune responses. IL-17 production has also been documented in patients infected with COVID-19 (Zumla et al., 2020). Macrolid antibiotics, including azithromycin, have been shown to reduce the IL-17 responses and exhibit neutrophil-directed pleiotropic anti-inflammatory effects. Their promising prophylactic as well as therapeutic activity in animal models of severe acute lung injury and capillary leak caused by influenza virus as well as endotoxin was associated with a markedly improved survival outcome (Tamaoki et al., 1995; Sato et al., 1998; Leiva et al., 2008). In a prospective, observational cohort, and multicenter study conducted in 27 ICUs of nine European countries involving 218 consecutive patients requiring invasive mechanical ventilation in the context of a community-acquired pneumonia (CAP), the use of macrolide antibiotics, including azithromycin, significantly reduced mortality (Martin-Loeches et al., 2010). Azithromycin was associated with more ICU-free days in severe sepsis patients with and without pneumonia (Martin-Loeches et al., 2010; Afshar et al., 2016). Importantly, the analysis of data from the Acute Respiratory Distress Syndrome Network (ARDSNet) showed that in patients with ARDS, early use of macrolide antibiotic therapy is associated with a survival advantage and decreased time to successful discontinuation of mechanical ventilation (Walkey and Wiener, 2012).

The potent and pleiotropic anti-inflammatory effects of azithromycin have long been known to immunologists and leveraged in lung transplantation (LTx) settings as well (Vos et al., 2012; Vos et al., 2014; Ruttens et al., 2016; Van Herck et al., 2019; Vos et al., 2019). Bronchiolitis obliterans syndrome (BOS) is a major subtype of lung allograft dysfunction that develops in half of the adult LTx recipients. Approximately, 40% of patients with BOS respond to azithromycin owing to its immunomodulatory and anti-inflammatory effects (Vos et al., 2012). Azithromycin reduces and prevents bronchoalveolar lavage (BAL) neutrophilia after LTx. Verleden et al. reported that CD8+ cells were the major source of IL-17 during lymphocytic bronchiolitis in LTx patients and azithromycin significantly reduced the IL-17 response (Verleden et al., 2013). Vos et al. investigated the clinical potential of azithromycin for the treatment of lymphocytic airway inflammation after LTx (Vos et al., 2014). Fifteen lung transplant recipients demonstrating acute allograft dysfunction due to isolated lymphocytic airway inflammation were prospectively treated with azithromycin for at least 6 months (ClinicalTrials.gov Identifier: NCT01109160). Administration of azithromycin was associated with suppression of posttransplant lymphocytic airway inflammation and clinical improvement in lung allograft function (Vos et al., 2014) The anti-inflammatory effects of azithromycin were recently confirmed in a controlled randomized clinical trial (ClinicalTrials.gov Identifier: NCT01915082) (Ruttens et al., 2016). Prophylactic azithromycin treatment has been demonstrated to improve freedom from BOS 2 years after LTx (ClinicalTrials.gov Identifier: NCT01009619) (Ruttens et al., 2016). Post-azithromycin use of the leukotriene inhibitor montelukast further improved the treatment outcomes (Vos et al., 2019). Notably, azithromycin was found to amplify the clinical benefit of hydroxychloroquine in a recently completed open label study in COVID-19 patients (Gautret et al., 2020).

Interleukin 6 (IL6) is a pro-inflammatory cytokine that plays a pivotal role in the development, progression, and severity of CRS as well as its complications, including disseminated intravascular coagulopathy (DIC) and multiorgan failure (The National Heart, Lung, and Blood Institute Acute Respiratory Distress Syndrome (ARDS), 2006; Teachey et al., 2013; Garcia Borrega et al., 2019; Khadka et al., 2019). In patients with lymphoid malignancies developing severe CRS after treatment with CD19-directed CAR-T cells such as tisagenlecleucel or the bispecific T-cell engager blinatumomab, blocking IL6 signaling by using a monoclonal antibody to the IL6 receptor (e.g., tocilizumab and siltuximab) was highly effective leading to a rapid resolution of the CRS (Teachey et al., 2013; Davila et al., 2014; Maude et al., 2014; Khadka et al., 2019). In 2017, tocilizumab (ACTEMRA) was approved by the FDA for the treatment of tisagenlecleucel-induced CRS. Early data from a single-arm, 21-patient Chinese trial indicated that an anti-IL6 receptor monoclonal antibody may have significant clinical benefit in COVID-19 pneumonia patients and China has approved the use of that antibody in severe forms of COVID-19. In an open label Phase 2 study (ClinicalTrials.gov Identifier: NCT04317092), Tocilizumab is being evaluated in patients with COVID-19 pneumonia and in a related randomized study (ClinicalTrials.gov Identifier: NCT04306705), it is being evaluated for its efficacy in CRS associated with COVID-19. It is also being evaluated in combination with Favipiravir (ClinicalTrials.gov Identifier: NCT04310228). Likewise, Sarilumab (Kevzara), another monoclonal antibody that binds to the IL-6 receptor, is being evaluated in a Phase 2/3 study in hospitalized COVID-19 patients (ClinicalTrials.gov Identifier: NCT04315298).

CRS can also be associated with a clinical picture (i.e., high fevers, hepatosplenomegaly, liver dysfunction, DIC, and hyperferritinemia) consistent with hemophagocytic lymphohistiocytosis (HLH) or macrophage activation syndrome (MAS) requiring a short pulse therapy with high doses of steroids. Targeted therapeutic agents of interest include Emapalumab (Gamifant), an FDA-approved monoclonal antibody that binds and neutralizes interferon gamma and Lenzilumab, an investigational monoclonal antibody that binds and neutralizes GM-CSF. A strong association between GM-CSF and severity of CRS has been observed in clinical trials (Khadka et al., 2019). In a recent press release, Humanigen announced that it wants to carry out a Phase 3 clinical trial for its lenzilumab to treat CRS in COVID-19 patients.

Activation of the complement signaling pathway by SARS-CoV infection contributes to the inflammation associated with the pathophysiology of ARDS in severe cases of SARS-CoV infection owing to production of chemoattractant complement C3a, C4, and C5a that contribute to recruitment and activation of neutrophils (Wang et al., 2015; Gralinski et al., 2018). In preclinical models of MERS, treatment of MERS-CoV–infected mice with C3a receptor antagonists or anti-C5a antibodies reduces acute lung injury and lung inflammation. Furthermore, complement-deficient mice have reduced neutrophilia in their lungs and reduced systemic inflammation (Gralinski et al., 2018). The complement inhibitor Eculizumab (Soliris), an FDA and EMA-approved humanized monoclonal antibody that binds to the complement component C5, is being used in an attempt to reduce the mortality in COVID-19 patients in the SOLID-C19 Expanded Access study (ClinicalTrials.gov Identifier: NCT04288713). It is also noteworthy that the recombinant human anti-vascular endothelial growth factor (VEGF) antibody Bevacizumab has entered clinical testing in COVID-19 patients with severe or critically severe pneumonia (BEST-RCT, ClinicalTrials.gov Identifier: NCT04305106) because of the potential contribution of VEGF to capillary leak in the lungs of patients with ARDS (Rosa, 2020).

The insights and lessons learned from clinical trials of anti-leukemia protocols with CRS-causing therapies indicate that combinations of tocilizumab, infliximab, and steroids or even chemotherapy with etoposide or cyclophosphamide may be necessary in management of severe cases with concomitant HLHI/MAS. The demonstrated clinical activity of Azithromycin therapy resulting in reduced inflammation and improved survival outcome in various preclinical and clinical settings of pneumonitis and ARDS, warrants its early institution in COVID-19 patients irrespective of the anti-viral agent that is being used before they develop signs of CRS or ARDS. Other interventions such as the use of an anti-IL6R antibody alone or in combination of other immune-modulatory agents of interest that block GM-CSF or complement signaling could be added to azithromycin. Adaptive clinical trials with parallel enrollment to different arms each evaluating a rationally designed combination modality could provide the foundation for the accelerated identification of effective and safe multi-modality treatment algorithms for COVID-19 pneumonia. There has been an increasing level of concern regarding potential short-term and long-term toxicities of chloroquine, hydroxychloroquine, and azithromycin in the management of COVID-19, including prolongation of the QTc interval, hypoglycemia, neuropsychiatric effects, and severe cutaneous adverse reactions, including Stevens-Johnson syndrome, toxic epidermal necrolysisretinopathy, vacuolar myopathy, neuropathy, restrictive cardiomyopathy, and cardiac conduction disturbances (Juurlink, 2020).

## Author Contributions

FU conceived the review, analyzed the contents of relevant publications, and wrote the manuscript. No medical writer or editor was involved.

## Conflict of Interest

Author FU was employed by the company Worldwide Clinical Trials.
